# Information Entropy Suggests Stronger Nonlinear Associations between Hydro-Meteorological Variables and ENSO

**DOI:** 10.3390/e20010038

**Published:** 2018-01-09

**Authors:** Tue M. Vu, Ashok K. Mishra, Goutam Konapala

**Affiliations:** Glenn Department of Civil Engineering, Clemson University, Clemson, SC 29634, USA

**Keywords:** information entropy, mutual information, kernel density estimation, ENSO, nonlinear relation

## Abstract

Understanding the teleconnections between hydro-meteorological data and the El Niño–Southern Oscillation cycle (ENSO) is an important step towards developing flood early warning systems. In this study, the concept of mutual information (*MI*) was applied using marginal and joint information entropy to quantify the linear and non-linear relationship between annual streamflow, extreme precipitation indices over Mekong river basin, and ENSO. We primarily used Pearson correlation as a linear association metric for comparison with mutual information. The analysis was performed at four hydro-meteorological stations located on the mainstream Mekong river basin. It was observed that the nonlinear correlation information is comparatively higher between the large-scale climate index and local hydro-meteorology data in comparison to the traditional linear correlation information. The spatial analysis was carried out using all the grid points in the river basin, which suggests a spatial dependence structure between precipitation extremes and ENSO. Overall, this study suggests that mutual information approach can further detect more meaningful connections between large-scale climate indices and hydro-meteorological variables at different spatio-temporal scales. Application of nonlinear mutual information metric can be an efficient tool to better understand hydro-climatic variables dynamics resulting in improved climate-informed adaptation strategies.

## 1. Introduction

For many water resources planning and management studies, reliable preliminary estimates of dependence between two hydroclimatic variables are extremely important. For example, knowledge of dependence between large-scale climate patterns such as El Niño–Southern Oscillation (ENSO) [1], the Pacific Decadal Oscillation (PDO) [2], and the Atlantic Multi-decadal Oscillation (AMO) [3] with local precipitation, temperature, or streamflow has resulted in improved longer lead-time forecasting models [4,5,6]. Large-scale climate patterns can also predict ecological processes better than local weather [7]. In addition to that, several studies indicated the presence of a significant relationship between large-scale climate phenomena and hydrologic extremes, such as extreme precipitation events [8,9,10], droughts [11,12,13], and floods [14,15]. Therefore, the presence of any kind of significant dependence forms the preliminary metric to identify appropriate predictors for forecasting streamflow and other hydroclimatic variables in ungauged river basins [16,17,18]. As a result, the predictability of these large-scale climate patterns much in advance is extremely important to improve the design of early warning systems of extreme events [19].

A wide range of methods is available for detecting the presence of an association between bivariate data set. Among them, Pearson’s correlation coefficient is the most widely used metric to quantify the linear dependence between any two variables [20]. Pearson correlation coefficient is based on the assumption that the considered variables follow a Gaussian distribution. Therefore, using Pearson correlation in case of variables that follow non-Gaussian distributions may be suboptimal. 

However, several insights were derived from these linear associations. For example, Zhang et al. [21] quantified the Pearson linear correlation between different sea surface temperature (SST) anomalies and seasonal precipitation for Huai River basin in China. The authors identified some positive/negative correlation with the coefficients ranges from absolute 0.2 to 0.3. Whereas, Wrzesinski [22] characterized and confirmed the strong influences of large-scale climate index NAO to seasonal river flow in Poland by comparing the difference in average runoff during the positive and negative NAO phases. On the same lines, Wrzesinski [23] also found the linkage between NAO and European river streamflow at the 140 gauging station by analyzing the high and low water flows according to the positive and negative NAO phases. 

In addition, most of the hydro-climatic series do not follow a Gaussian distribution leading to a possible misinterpretation of the dependency between the variables when using linear measures [24,25,26]. As a result, the non-parametric rank-based correlation metrics of Kendall’s tau and Spearman’s rho are applied to quantify the relationship between two given variables [27,28,29]. However, even though the associations evaluated by these two metrics are independent of any probability distribution assumptions, they are more successful in detecting the monotonous relationships between any two variables. In addition, assuming a monotonous relationship among two hydroclimatic variables might be too restrictive to characterize the existing complex dependence structures between the hydroclimatic variables [30,31,32]. 

To overcome the limitations of linear dependence, several studies in hydro-climatology adopted the concepts based on nonlinear statistics to evaluate the strength of association among distinct hydroclimatic variables. For instance, Fleming et al. [33] observed a nonlinear association between northern hemisphere river basin streamflow and teleconnection patterns. In regard to climate extremes, Cannon [34] utilized generalized extreme value distributions to investigate the relationship between ENSO and winter extreme station precipitation in North America. Whereas Lin-Ye et al. [35] investigated the relationship between extreme events of wave storms and large-scale climate covariates using generalized additive and linear models. While Kusumastuti [36] studied the nonlinearity between threshold and rainfall-runoff transformation. More recently, Konapala et al. [37] have investigated the nonlinear relationship between low flows in Texas river basins with large scale climatic patterns. 

Recently entropy-based approaches are gaining popularity for different applications, such as, climate variability [38], uncertainty analysis [39], hydrometric network analysis [40,41,42], selection of predictors [43,44], and drought regionalization [45]. Similarly, the entropy theory based mutual information (*MI*) is widely used for quantifying the non-linear association between multiple variables [40,41]. The *MI* concepts have been utilized to quantify nonlinear dependence structure between several climate variables across the globe [46,47]. Also, several *MI* metrics were developed to assist in predictor selection for hydroclimatic forecasting [48,49]. More importantly, *MI* was able to detect the presence of strong nonlinear associations of streamflow, rainfall, and global mean temperature with several large-scale climatic variables [31,50,51,52,53,54].

Unlike the signals in other research fields, hydroclimatic data being a subset of geophysical research field are non-repeatable, shorter in length and contaminated with significant noise levels [31]. In addition to that, studies have indicated a presence of chaotic component [55]. Therefore, it becomes evident that the selected measure should be relatively robust to noise and chaos and, more importantly, detects signals possibly shorter in length. Thus, the goal of this study is to investigate and compare the performance of nonlinear estimation method based on mutual information (*MI*) with the traditional linear association metric of Pearson correlation. For this purpose, the hydro-meteorological data are considered such as total annual precipitation, annual average streamflow, and extreme precipitation indices specific to Mekong River Basin in Southeast Asia and estimated its association with the large-scale climate index of ENSO. 

## 2. Materials and Methods 

### 2.1. Hydro-Meteorological Data and ENSO

**Figure 1:**
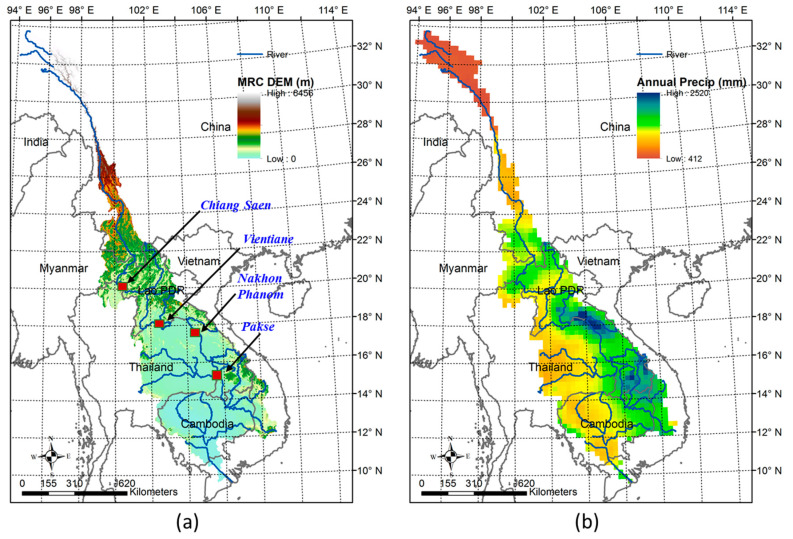
(**a**) Mekong River Basin map with digital elevation model terrain and digitized river, location of four hydro-meteorology stations are displayed; (**b**) Annual rainfall distribution over MRB using APHRODITE gridded rainfall 1951–2007.

The Mekong River Basin (MRB), originates from Tibetan Plateau in China and flows through the territory of five other countries in the Southeast Asia (Myanmar, Laos PDR, Thailand, Cambodia, and drains to the sea in Vietnam) (Figure 1a). This is currently home to more than 70 million people and it is expected to increase by 100 million in 2050 [56]. The detailed topography map of study region is displayed in Figure 1a with relative high terrain in the upstream of the basin (upper Mekong). In this study, the gridded precipitation data is provided from Asian Precipitation Highly Resolved Observational Data Integration Towards the Evaluation of Water Resources (APHRODITE) with a spatial resolution of 0.25° and daily temporal resolution with the sufficiently long record period from 1951–2007 covering the Asian monsoon domain. The dataset was created primarily with data obtained independently from rain gauge observation network across all the regions of Asia. The daily precipitation values from rain gauges were interpolated using the sphere map technique [57] and the first six components of the fast Fourier transforms were taken to obtain daily data for all land areas in the Asian monsoon domain [58]. This dataset is referred to as “APH” in this paper, for the purpose of brevity. APH has been previously utilized as a reliable gridded rain data set over this area for various hydrological applications [59,60,61,62,63]. The spatial distribution of total precipitation from APH data over MRB is displayed in Figure 1b with high rainfall volumes (around 2000 to 2500 mm) gathering around the eastern part of the river basin near to the Annamite mountain range. 

**Figure 2:**
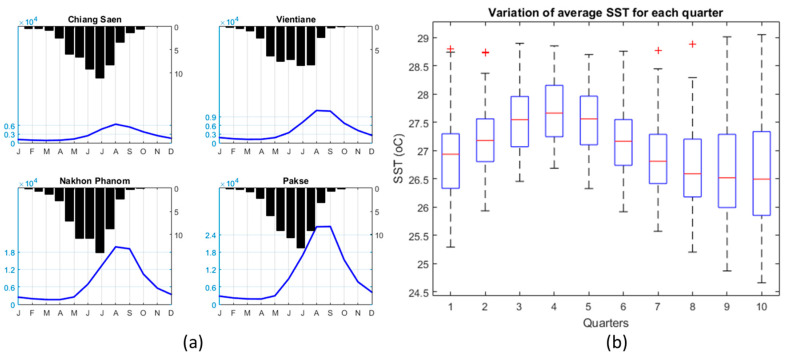
(**a**) Seasonal cycle of monthly climatology rainfall and streamflow measured at four stations on the mainstream Mekong river; (**b**) Boxplots of monthly SST Nino 3.4 for different quarters

The southwest monsoon wind originated from the Gulf of Thailand blocked by the Annamite mountain range is the main factor that causes the high seasonal rainfall in summer months (June, July August, September) [61] (Figure 2a). The APH daily gridded rainfall values were extracted to station locations using bilinear interpolation [59,63,64] to four meteorological stations located in MRB—Chiang Saen, Vientiane, Nakhon Phanom, and Pakse (Figure 1a)—for the time period of 1951 to 2007 over the Mekong River Basin. In addition to rainfall data, the corresponding annual streamflow data were also collected at these four hydrological stations from the MRC website for the same period. The monthly mean climatology over 57 years of hydro-meteorological stations is displayed in Figure 2a. Even though different patterns are found over four stations, the hydrographs are quite similar from the upstream station at Chiang Saen to downstream station Pakse, with flooding season from May to November, while the peak discharge months are around August and September. This study considers the seasonal cycle which includes all 12 months of the hydrologic year (starting from the month with the lowest rainfall—i.e., May—and ending in April of the next year; the streamflow month is considered to lag one month which starts from June and ends in May next year).

The study focuses on the total annual rainfall, annual streamflow, as well as extreme precipitation indices [65] for hydrologic years derived from daily precipitation datasets for both wet and dry spell statistics, which are (1) R5d: Max consecutive five-day rainfall, (2) P90p: 90th percentile of the daily precipitation time series for the year, (3) SDII: average daily precipitation on a wet day, and (4) dry spell length computed from daily precipitation for a year. Among four stations, Pakse has the highest streamflow data due to its furthest downstream location. Nakhon Phanom and Pakse have the highest rainfall as seen in Figure 1 and Figure 2.

The assumption has been that the long-term hydro-meteorological data variability can be captured by the fluctuation of the SST over seasonal scales. In this study, the ENSO index has been considered by using the SST over NINO 3.4 region [60,66], which is the area averaged monthly SST over the region bounded by the coordinates 5° N–5° S, 170° W–120° W. This time series could effectively indicate the occurrence of ENSO events [66]. The dataset can be downloaded from the Climate Data Guide website by NCAR [67]. Therefore in this study, the ENSO indices are computed at multiple three-month time windows in terms of quarters [31] consisting of the average SST anomalies over months of JFM, FMA, MAM, etc., for all the years (J: Jan, F: Feb, M: Mar). The SST over Nino 3.4 regions boxplots bound for 57 years for 10 different quarters are displayed in Figure 2b. The red crosses in Figure 2b denote outliers. There are four quarters just before the seasonal cycle, four quarters corresponding to the seasonal cycle, and two quarters after seasonal cycle, computed with the mean monthly SST over Nino 3.4 region. Among all 10 quarters, the fourth quarter (AMJ) has the highest median and whisker values compared to other quarters whilst the last two quarters (9 and 10) have the lowest median values.

### 2.2. Mutual Information Estimation

The mutual information (*MI*) has been utilized to capture the nonlinear dependence structure between two random variables. When analyzing experimental times series from the non-linear system, the *MI* is especially an important statistics [68]. According to [69], there are three theorems for *MI* between two random variables *X*, *Y*: (1) *MI* is non-negative and is zero if *X* and *Y* are strictly independent; (2) *MI* is infinity if there exists a function “g” such that *X* = g(*Y*); (3) *MI* is invariant to separate one to one transformations.

The *MI* can be computed using the relative entropy suggested by Joe [70]. Assuming that a pair of continuous random variables (*X*, *Y*) exist which have a joint probability density function (pdf) $p_{XY}$ and with its marginal pdf accordingly $p_{X}$ and $p_{Y}$. The mutual information or relative entropy can be defined as

(1) $$MI(X,Y)=\int \int p_{XY}\log\frac{p_{XY}}{p_{X}p_{Y}}dxdy$$

It is noted that the Equation (1) measures the distance between a joint distribution and the distribution when there is independence [69]. In case of continuous variables, there is no direct way to accurately determine continuous probability distributions. Therefore, several methods have been introduced to approximate the continuous probability distribution functions as discrete distributions. Among them, Khan et al. [71] compared four different methods to estimate the probability distribution function to calculate *MI* using: kernel density estimator (KDE) [68], K-nearest neighbors (KNN) [72], Edgeworth approximation of multivariate differential entropy [73], and adaptive partitioning of the *XY* plane [74]. The authors found that KDE and KNN outperform the other two methods in term of their ability to capture the dependence structure. Khan et al. [31] indicated that KDE is able to capture the underlying nonlinear dependence more consistently compared to KNN and Edgeworth when they are short and noisy assuming such dependence exists. Therefore, this article utilizes the KDE to estimate probability density function using the equation

(2) $$p\left(x\right)=\frac{1}{n}\sum_{i}^{n}K\left(u\right)$$

in which, *K*(*u*) is a multivariate kernel function

(3) $$K\left(u\right)=\frac{1}{h^{d}\sqrt{{\left(2\pi \right)}^{d}\det(S)}}\exp\left(-\frac{u}{2}\right)$$

(4) $$u=\frac{{\left(x-x_{i}\right)}^{T}S^{-1}\left(x-x_{i}\right)}{h^{2}}$$

From (4), *x_i_* a *d*-dimensional random vector for the multivariate data set (*x*_1_, …, *x_n_*). *S* is the covariance matrix on the *x_i_*, det(*S*) is a determinant of *S* and *h* is the kernel bandwidth or smoothing variable. The optimal Gaussian bandwidth “*h*” can be computed using Equation (5) [75]

(5) $$h={\left(\frac{4}{d+2}\right)}^{\frac{1}{d+4}}n^{-\frac{1}{d+4}}$$

The “*d*” is taken from [76] for the value of *d* = 2, and is similar to [68].

Substituting Equations (3) and (4) to Equation (2) to obtain the approximate probability density function as

(6) $$p\left(x\right)=\frac{1}{n}\sum_{i}^{n}\frac{1}{h^{d}\sqrt{{\left(2\pi \right)}^{d}\det(S)}}\exp\left[-\frac{{\left(x-x_{i}\right)}^{T}S^{-1}\left(x-x_{i}\right)}{2h^{2}}\right]$$

The detailed procedure using KDE to estimate pdf can be found in [31,68,71]. Here, the discrete formulation of *MI* is shown in Equation (7)

(7) $$MI(X,Y)=\frac{1}{n}\sum_{i=1}^{n}\ln\frac{p_{XY}\left(x_{i},y_{i}\right)}{p_{X}(x_{i})p_{Y}(y_{i})}$$

In which $p_{XY}\left(x_{i},y_{i}\right)$ is the joint pdf and $p_{X}\left(x_{i}\right),p_{Y}\left(y_{i}\right)$ are marginal pdfs at $\left(x_{i},y_{i}\right)$. The *MI* values range between independent (*MI* = 0) to completely dependent (*MI* = ∞). In order to make the generalization of the correlation with a range from 0 (independent) to 1 (dependent), Joe [70] proposed a formula to transform the *MI* to nonlinear correlation coefficient (*NLCC*)

(8) $$NLCC=\sqrt{\left[1-\exp\left(-2MI\right)\right]}$$

The *NLCC* range is similar to linear correlation coefficients (*LCC*) and has been used in most of the studies by [31,69,70]. 

**Figure 3:**
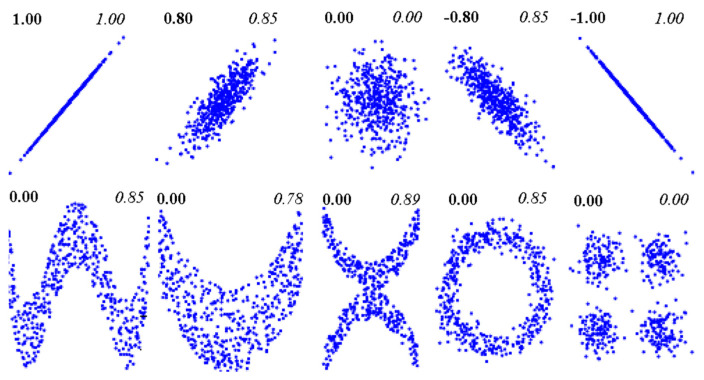
The comparison between linear correlation coefficient (*LCC*) and nonlinear correlation coefficient (*NLCC*) obtained from mutual information (*MI*) computed based on two random variables. For each panel, top left value (bold) shows *LCC* and top right (italic) represents *NLCC*. The top row illustrates the similarity between *LCC* and *NLCC*, whereas, the bottom row exhibits advantages of non-linear relationship based on different distribution. (Figures partially adopted from [77] using their python code. The *NLCC* was computed based on MI values).

Figure 3 modified [77] using their python code to demonstrate the *LCC* and *NLCC* using the scatter plots of two random variables obtained from a different sample of data. The advantage of *NLCC* is based on the fact that *MI* makes no assumption on the distribution of the variables or the nature of the relationship between them and is sensitive to nonlinear and non-monotonic effects [77]. It can be observed that (top row of Figure 3) both the *LCC* and *NLCC* can able to capture the linear relationship between the two random variables with a very close range of values. However, the advantage of *NLCC* compared to *LCC* is due to its ability to recognize the different distributions of the two random variables (bottom row of Figure 3).

Subsequently, the *LCCs* are computed between each of the precipitation indices and compared with *NLCC*s at four hydro-meteorology stations located in MRB (Figure 1a). The four stations are chosen based on their differences in terms of seasonal cycles, total annual rainfall amount and geographical height. Finally, the gridded linear/non-linear CC is constructed for the MRB to showcase the spatial variability of the correlation coefficient.

## 3. Results

### 3.1. Linear and Nonlinear Correlation between Annual Precipitation/Streamflow and ENSO Index

**Figure 4:**
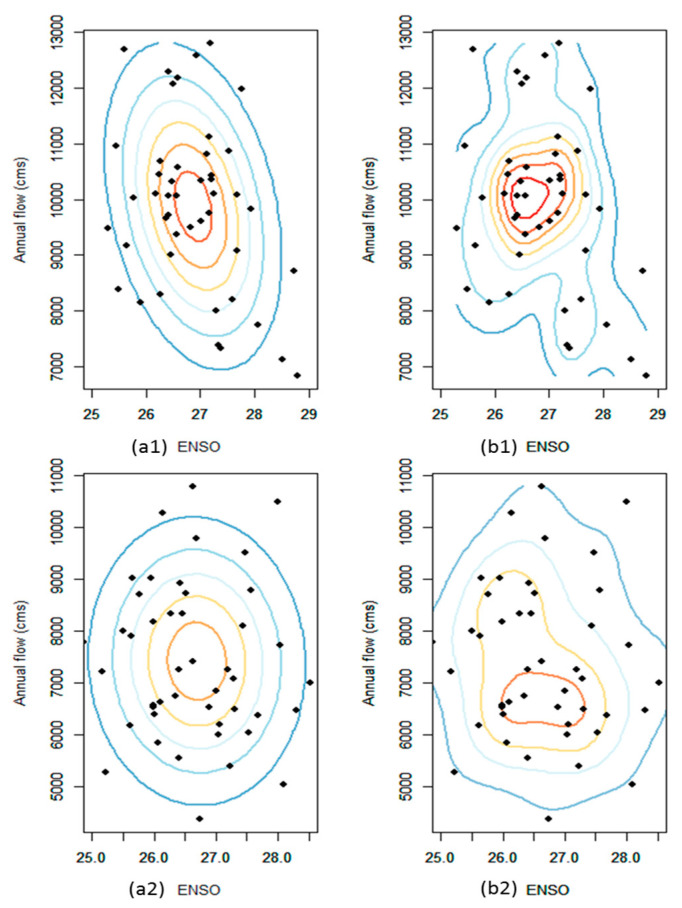
The bivariate normal and kernel density between annual flow and ENSO index for (**a**) normal density; (**b**) kernel density; (1) Quarter 1 at Pakse station (highest linear and nonlinear CCs) (2) Quarter 9 at Nakhon Phanom station (lowest, respectively).

We first illustrate the linear dependence as a bivariate normal distribution and nonlinear dependence as a kernel bivariate distribution following the work of Khan et al. [71]. The bivariate normal and kernel density between the annual average streamflow at different hydrologic gauging stations and different quarters of ENSO indices are computed and plotted in Figure 4 for the highest and lowest linear and nonlinear CCs. For kernel density, a Gaussian kernel with an optimal Gaussian bandwidth computed by $h=N^{-1/6}$ with *N* is the total number of observed points (57 in this case).

**Figure 5:**
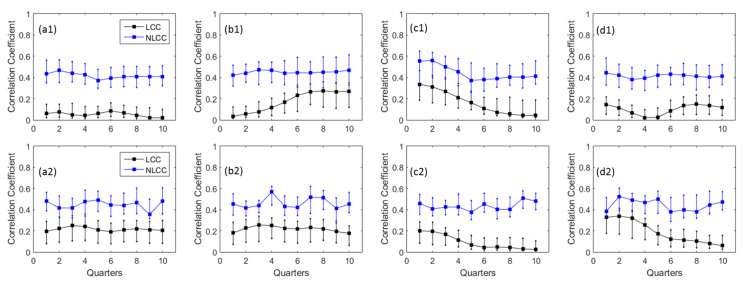
Linear and nonlinear CCs with their 90% confidence intervals between SST Nino 3.4 and (1) total annual precipitation (2) annual average streamflow for (**a**) Chiang Saen; (**b**) Vientiane; (**c**) Nakhon Phanom; and (**d**) Pakse. Note: The correlation coefficients (*CCs*) are calculated between precipitation and ENSO indices in-terms of quarterly temporal windows, such as, JFM, FMA, and MAM and so on. The 90% confidence intervals are computed using 100 simulations by bootstrapping approach.

Subsequently, the linear and nonlinear CCs between total annual precipitation and average streamflow were computed at different window lengths of SST Nino 3.4 (quarters) for four different hydro-meteorology stations and display in Figure 5. Based on the formulation Equation (8) *NLCCs* are positive, therefore the absolute value of *LCCs* are computed for comparison plotting in Figure 5 and all other figures henceforth. In order to compute the 90% confidence intervals for the absolute correlation coefficients, the bootstrapping approach [71] was applied using 100 simulations and plotted in Figure 5. It can be seen in Figure 5 that the *NLCC*s have higher values than the *LCCs* for all quarters. It indicates that the KDE captures the more extrabasinal connection between ENSO and precipitation as well as river flows compared to linear modeling [31].

In particular at station scale, Nakhon Phanom has the highest precipitation amount, its *LCCs* and *NLCC*s are also among the highest for the first four quarters Figure 5(c1,c2) which subsequently decrease. The *NLCC* values also have the same patterns as *LCCs* for this station as well as Pakse in Figure 5(d1). Although both stations have highest CCs for the first quarter, however, the lowest CCs values are not found at the same: Quarter 7 (Nakhon Phanom) and Quarter 4 (Pakse). Chiang Saen and Vientiane have variation trends for *LCCs* Figure 5(a1,b1) among all quarters, this is perhaps due to the less rainfall amount observed at these two rain gauges. 

Among four hydrology gauging stations, Pakse also has highest *LCCs*/*NLCC*s with ENSO at the first four quarters Figure 5(d2). This is because Pakse is the furthest station downstream and it has the highest streamflow measured. Therefore, it shows more dependences by the fluctuations of ENSO indices. The second highest streamflow station is Nakhon Phanom (see Figure 5(c2)) that also indicates similar patterns of *LCCs* and *NLCCs* with higher dependences with the first four quarters of ENSO indices compared with the rest. When the *LCC* values are close to zero (as in the last four quarters of Figure 5(c2)), the differences between *LCCs* and *NLCCs* should be interpreted with caution because of the exponential scaling of *MI* in *NLCC* as shown in Equation (8). The two upstream gauging stations Chiang Saen and Vientiane (Figure 5(a2,b2)) also have similar patterns for *LCCs* and *NLCC*s at different quarters. Compared to total annual precipitation, the annual average streamflow has slightly higher *LCCs* and *NLCCs*, except for Nakhon Phanom station. Overlapping the 90% confidence intervals of *LCCs* and *NLCCs* for streamflow at Chiang Saen, Vientiane, and Pakse (Figure 5(a2,b2,d2)) for the first three quarters indicates that both KDE and linear regressions effectively capture the strong dependence structure. 

### 3.2. Linear and Nonlinear Correlation between Precipitation Extreme Indices and ENSO

**Figure 6:**
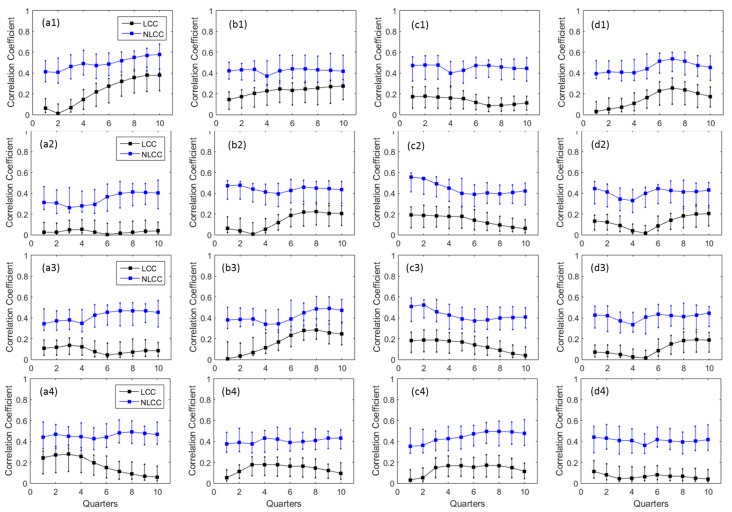
Comparison between linear and nonlinear *CCs* derived between SST Nino 3.4 and (1) R5d; (2) P90p, (3) SDII; (4) dry spell for selected stations located at (**a**) Chiang Saen; (**b**) Vientiane; (**c**) Nakhon Phanom; and (**d**) Pakse. Note: The correlation coefficients (*CCs*) are calculated between precipitation and ENSO indices in-terms of quarterly temporal windows, such as JFM, FMA, MAM, and so on. The 90% confidence intervals are computed using 100 simulations by bootstrapping approach.

In addition to total annual precipitation and streamflow dependences, further investigations on *LCCs* and *NLCCs* are carried out between precipitation indices for extreme values indices (R5d, SDII, P90p, dry spell) with different quarters of ENSO events. This analysis aims to quantify the dependence structure between annual extreme precipitation events and ENSO indices. The wet indices (R5d, P90p, SDII), as well as dry indices (dry spell), are also taken into account for the analyses. The detail dependences based on *LCCs* and *NLCCs* can be found in Figure 6 for four indices and four stations over 10 quarters. Similar to Figure 5, the *LCCs* are displayed as absolute values comparable to *NLCCs.* The 90% confidence intervals for the absolute correlation are computed based on 100 simulations using bootstrapping approach. The detail analyses on Figure 6 reveals several significant dependencies between annual extreme precipitation events and ENSO indices. Chiang Saen exhibits high *LCCs/NLCCs* for a maximum five consecutive days of rainfall with the last three quarters of ENSO indices and low *CCs* values for the first three quarters Figure 6(a1). Similar patterns are found for Pakse station in Figure 6(d1) but with the highest *CCs* among Quarters 6, 7, 8 and lowest at the first three quarters. This variation is different from the total annual precipitation in Figure 5(a1,d1). In contrast to R5d, the other two indices: P90p and SDII have similar patterns of *LCCs/NLCCs* compared to the total annual precipitation in Figure 5. The last three-quarters of ENSO indices show that it has higher *LCCs/NLCCs* for Vientiane and Pakse Figure 6(b2,b3,d2,d3) compared to other quarters whilst the first three quarters of Nakhon Phanom has the highest values than the last three quarters. The dry spell indices of all four stations show nearly opposite patterns compared to R5d. The overall pattern illustrates that nonlinear *CCs* have higher values (more dependencies) than linear *CCs* for all stations/indices, similar to Figure 5 and [31].

The analyses with total annual precipitation, annual average streamflow, and extreme precipitation indices on wet and dry conditions reveal that there exists a nonlinear extrabasinal connection between ENSO and the hydro-meteorological data over Mekong river basin. The analyses based on *LCCs/NLCCs* exhibit the increasing trend in the variation of annual statistics on hydro-meteorology in connection with ENSO by computing nonlinear relationship as compared to linear measures. Therefore, it is expected to give additional support for early prediction (compared to traditional linear measurement) based on the ENSO forecast with the hydro-meteorology connection when *MI*-based approaches are utilized. This approach, somehow, would be helpful in water resources management for drought mitigation, flood control, as well as an irrigation system for agricultural areas.

### 3.3. Spatial Linear and Nonlinear Correlation Maps

**Figure 7:**
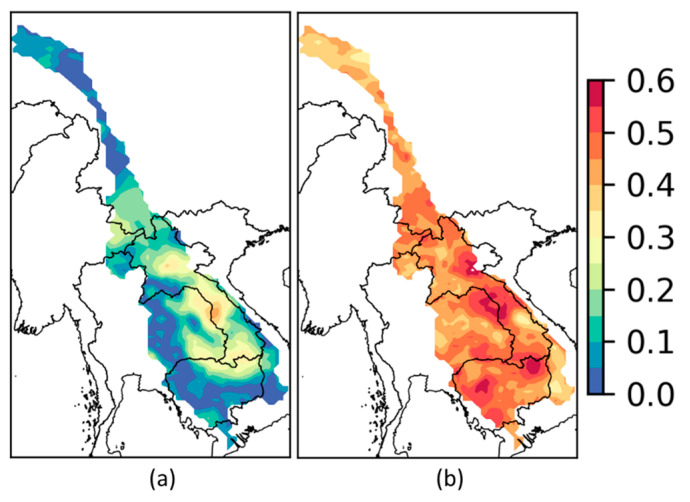
(**a**) Linear and (**b**) nonlinear CCs between Nino 3.4 at Quarter 1 and annual precipitation.

Based on the correlation analysis at four rainfall stations, it was observed that the *NLCCs* have higher values in comparison to *LCCs* at all the selected stations located in MRB. Further analyses of the spatial pattern using the gridded data from APH based on the selected statistics of annual precipitation and extreme indices have been carried out. The linear and nonlinear *CCs* between ENSO Quarter 1 and annual rainfall are displayed in Figure 7 (using the same color bar), and the correlation values range from 0 to 0.6. Quarter 1 was selected arbitrarily as the first quarter, even though, it shows the highest CCs over Nakhon Phanom and Pakse for total annual precipitation but it also displays the lowest CCs for other analyses such as for Vientiane extreme indices (Figure 6b) or Pakse in Figure 6(d1,d3). Figure 7a exhibits the higher *CC* values between total annual precipitation and ENSO Quarter 1 (about 0.4) from Nakhon Phanom to Pakse station, along with Laos, eastern Thailand, and northern Cambodia. This can be clearly observed during the first quarter time frame Figure 5(c1,d1). On the other hand, the *NLCCs* have higher *CC* values (about 0.5 to 0.6) for the same locations compared to other grids. The use of spatial distribution map can be extended to generate the teleconnection patterns in ungauged regions. That, in turn, helps to better inform local stakeholders in building better tools for water resource management.

**Figure 8:**
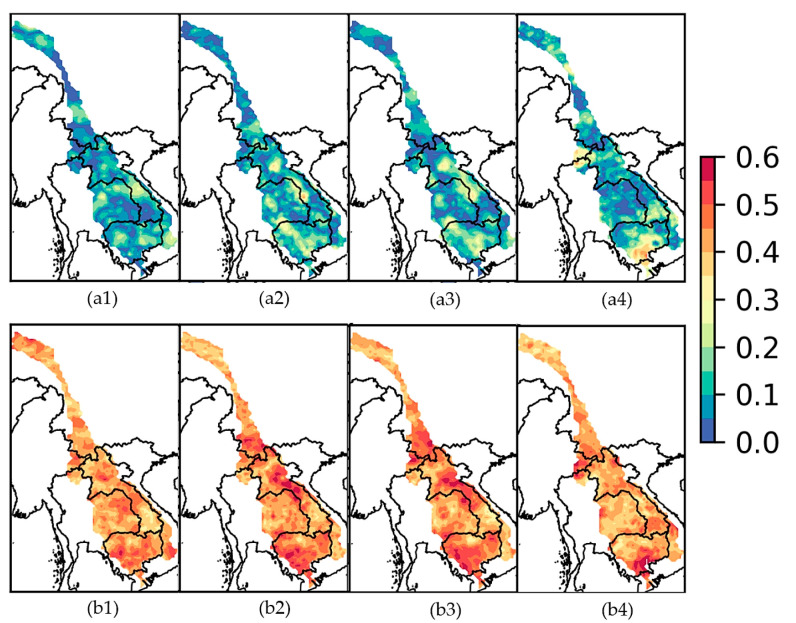
(**a**) Linear and (**b**) nonlinear CCs between Nino 3.4 at quarter 1, and (1) R5d, (2) P90p, (3) SDII, (4) dry spell.

Similarly, the dependency between selected precipitation indices (R5d, P90p, SDII, and dry spell) and Quarter 1 of ENSO are computed and displayed in Figure 8. The linear *CCs* obtained from precipitation indices (Figure 8) are slightly lower than that of annual precipitation in the previous analysis (Figure 7) for Laos, eastern Thailand, and northern Cambodia. There is slightly higher *LCCs/NLCCs* magnitude observed at southern Cambodia during a dry spell Figure 8(a4,b4). The magnitudes of *NLCC*s (Figure 8) are also slightly lower than *NLCC*s based on the annual precipitation (Figure 7). However, the values obtained from *NLCC*s are still significantly higher than *LCCs* for all extreme indices. The lower Mekong basin (the southern part of river basin spread over Laos, Thailand, and Cambodia to Vietnam) has higher correlations to ENSO indices compared to the northern/upper basin, similar to [78].

## 4. Discussion 

Although ENSO has a direct influence on rainfall anomalies over the tropical and subtropical regions, only a portion of the variation in the annual flow of rivers located in these regions is associated with ENSO events [31]. This study discusses in detail the possible dependences between different quarters of ENSO indices and hydro-meteorological dataset over Mekong river basin. Other existing studies on MRB highlighted that the linear correlations between ENSO and streamflow over the selected locations in Chiang Saen, Vientiane, and Pakse are around −0.4 to 0.2 [78] and maximum linear correlations between ENSO and precipitation over several stations in Thailand are around −0.18 to 0.22 [79]. The above correlation figures are in line with the study that the maximum LCCs obtained are around 0.35 for both annual precipitation and streamflow as displayed in Figure 5. The results from mutual information derived *NLCC*s therefore suggests an additional approach to look into the dependence structure for multivariate hydro-meteorological data with the large-scale climate patterns. ENSO is a periodic climatic phenomenon with 3–7 years of the cycle and can be predicted several seasons in advances [80]. For instance, Ludescher et al. [81] were able to predict the likelihood of El Niño conditions in 2014 almost a year in advance indicating an improvement in our prediction capacity. In addition, other researchers [82,83] also revealed the intensification of ENSO related precipitation and El Niño frequency in the future due to the warming associated with an increase in greenhouse gas emission. Moreover, Ward et al. [15] indicated that the global flood risk exists during El Niño or La Niña years, or both, in basins spanning 44% of the land surface of the Earth. Therefore, the predictability of ENSO has significant implications and the ability to predict in advance might lead to better local water resources management towards developing more efficient flood and drought early warning systems.

This study limits to using only the kernel density estimation approach to estimate the mutual information values. However, there are several methods that have been used to compute the mutual information, such as K-nearest neighbors (KNN) [72], Edgeworth approximation of multivariate differential entropy [73] and adaptive partitioning of the XY plane [74]. The detailed comparisons among these estimator approaches were carried out in [71] for a different type of data such as linear, quadratic, periodic function, chaotic system. Khan et al. [71] concluded that KDE is the best choice for very short data (50–100 data points). Therefore, KDE approach was utilized to estimate the bivariate mutual information in this study. In addition to mutual information method, there are several approaches used to build the nonlinear relationship between multivariate. Lin-Ye et al. [35] applied the VGAM/VGLM to quantify the nonlinear relationship between storm components and large-scale climate indices (NAO and others) using Global Climate Model data via the regression coefficients that were used to build the location and scale parameters of their statistical model. Zhang et al. [84] investigated the nonlinear relationship by employing the concept of mutual information to evaluate the dependency between the normalized difference vegetation index (NDVI) and meteorological variables for the middle reach of the Hei river basin. Higher dependency between NDVI and coupled precipitation/temperature was observed for the desert area whilst for oasis region, groundwater is an important factor for driving vegetation growth. The authors utilized the mutual information as a method to classify the study region into a smaller area (desert, oasis, artificial oasis). 

The *NLCCs* and *LCCs* analysis between ENSO and hydro-meteorological data suggest that there exists a nonlinear correlation for the Mekong River Basin. This finding agrees well with a previous study [28] with a focus on river flow analysis over tropical and sub-tropical river basins. It can be observed that although the linear CCs can be 0, the smallest *NLCC*s can be around 0.3 to 0.4. This needs caution from an artifact of Equation (8) which scales nonlinear CCs exponentially with *MI* [31] when linear CCs are close to 0. It, however, does not affect the other values of CCs higher than 0.

## 5. Conclusions

This study analyses the possible influence of the large-scale climate index ENSO and hydro-meteorological data in the form of total annual precipitation, annual average streamflow, and extreme precipitation indices using linear and nonlinear correlation over the Mekong River Basin. The nonlinear correlation structure was computed based on mutual information approach using marginal and joint entropy. The kernel density estimation approach was selected among several other techniques to estimate the mutual information values as this approach works best for very short data length of 50 to 100 points. Nonlinear correlations were obtained by transforming the mutual information using Joe formula [70] and thus are comparable with absolute linear correlation coefficients. Bootstrapping approach based on 100 simulations was applied to find the confidence intervals for these absolute correlation coefficients. In-depth analysis was carried out at four hydro-meteorological stations located in the Mekong River Basin. The major conclusions that can be drawn from this study are listed below:

1. Nonlinear correlation is able to reveal the additional dependence structures between hydro-meteorological data over Mekong River Basin and ENSO indices.
2. Both linear and nonlinear correlation exhibits similar varying patterns among different ENSO quarters for most of the stations/indices.
3. The results reveal that higher correlation coefficients can be found using the nonlinear correlation coefficients in comparison to the traditional linear correlation analysis.
4. Spatial correlation structures for *LCCs* and *NLCCs* are also constructed based on extreme precipitation indices and ENSO. The use of spatial maps further complements our analyses based on a single station to other ungagged regions to better inform local stakeholders in building better tools for water resource management.
5. Further analyses are required to reveal the non-linear association between other large-scale climate phenomena (SOI, PDO, NAO, etc.) with local meteorological variables. The mutual information between these indices and local meteorological variables can further help policymakers to improve climate-informed adaptation studies.

## References

[1] Gu D., Philander S.G.H. (1997). Interdecadal climate fluctuations that depend on exchanges between the tropics and extratropics. Science.

[2] Mantua N.J., Hare S.R., Zhang Y., Wallace J.M., Francis R.C. (1997). A Pacific interdecadal climate oscillation with impacts on salmon production. Bull. Am. Meteorol. Soc..

[3] Enfield D.B., Mestas-Nuñez A.M., Trimble P.J. (2001). The Atlantic multidecadal oscillation and its relation to rainfall and river flows in the continental US. Geophys. Res. Lett..

[4] Wood A.W., Maurer E.P., Kumar A., Lettenmaier D.P. (2002). Long-range experimental hydrologic forecasting for the eastern United States. J. Geophys. Res. Atmos..

[5] Tootle G.A., Piechota T.C., Singh A. (2005). Coupled oceanic-atmospheric variability and US streamflow. Water Resour. Res..

[6] Kalra A., Ahmad S. (2009). Using oceanic-atmospheric oscillations for long lead time streamflow forecasting. Water Resour. Res..

[7] Hallett T.B., Coulson T., Pilkington J.G., Clutton-Brock T.H., Pemberton J.M., Grenfell B.T. (2004). Why large-scale climate indices seem to predict ecological processes better than local weather. Nature.

[8] Cayan D.R., Redmond K.T., Riddle L.G. (1999). ENSO and hydrologic extremes in the western United States. J. Clim..

[9] Jones C. (2000). Occurrence of extreme precipitation events in California and relationships with the Madden-Julian oscillation. J. Clim..

[10] DeFlorio M.J., Pierce D.W., Cayan D.R., Miller A.J. (2013). Western U.S. Extreme Precipitation Events and Their Relation to ENSO and PDO in CCSM4. J. Clim..

[11] Barlow M., Nigam S., Berbery E.H. (2001). ENSO, Pacific Decadal Variability, and U.S. Summertime Precipitation, Drought, and Stream Flow. J. Clim..

[12] Mo K.C. (2011). Drought onset and recovery over the United States. J. Geophys. Res..

[13] Özger M., Mishra A.K., Singh V.P. (2009). Low frequency drought variability associated with climate indices. J. Hydrol..

[14] Andrews E.D., Antweiler R.C., Neiman P.J., Ralph F.M. (2004). Influence of ENSO on flood frequency along the California coast. J. Clim..

[15] Ward P.J., Jongman B., Kummu M., Dettinger M.D., Weiland F.C.S., Winsemius H.C. (2014). Strong influence of El Niño Southern Oscillation on flood risk around the world. Proc. Natl. Acad. Sci. USA.

[16] Sivapalan M. (2003). Prediction in ungauged basins: A grand challenge for theoretical hydrology. Hydrol. Process..

[17] Hrachowitz M., Savenije H.H.G., Blöschl G., McDonnell J.J., Sivapalan M., Pomeroy J.W., Arheimer B., Blume T., Clark M.P., Ehret U. (2013). A decade of Predictions in Ungauged Basins (PUB)—A review. Hydrol. Sci. J..

[18] Samaniego L., Bárdossy A., Kumar R. (2010). Streamflow prediction in ungauged catchments using copula-based dissimilarity measures. Water Resour. Res..

[19] Chikamoto Y., Timmermann A., Luo J.J., Mochizuki T., Kimoto M., Watanabe M., Ishii M., Xie S.P., Jin F.F. (2015). Skilful multi-year predictions of tropical trans-basin climate variability. Nat. Commun..

[20] Hlinka J., Hartman D., Vejmelka M., Novotná D., Paluš M. (2014). Non-linear dependence and teleconnections in climate data: Sources, relevance, nonstationarity. Clim. Dyn..

[21] Zhang Q., Wang Y., Sing V.P., Gua X., Kong D.D., Xiao M.Z. (2016). Impacts of ENSO and ENSO Modoki+A regimes on seasonal precipitation variations and possible underlying causes in the Huai River basin, China. J. Hydrol..

[22] Wrzesinski D. (2011). Regional differences in the influence of the North Atlantic Oscillation on seasonal river runoff in Poland. Quaest. Geogr..

[23] Wrzesinski D. (2008). Typology of spatial patterns seasonality in European rivers flow regime. Quaest. Geogr..

[24] Lanzante J.R. (1996). Resistant, robust and non-parametric techniques for the analysis of climate data: Theory and examples, including applications to historical radiosonde station data. Int. J. Clim..

[25] Yue S., Pilon P., Cavadias G. (2002). Power of the Mann–Kendall and Spearman’s rho tests for detecting monotonic trends in hydrological series. J. Hydrol..

[26] Konapala G., Mishra A.K. (2016). Three-parameter-based streamflow elasticity model: Application to MOPEX basins in the USA at annual and seasonal scales. Hydrol. Earth Syst. Sci..

[27] Belle G., Hughes J.P. (1984). Nonparametric tests for trend in water quality. Water Resour. Res..

[28] Li J., Tan S. (2015). Nonstationary flood frequency analysis for annual flood peak series, adopting climate indices and check dam index as covariates. Water Resour. Manag..

[29] Zhang Y., Cabilio P., Nadeem K., Li W.K., Stanford D.A., Yu H. (2016). Improved Seasonal Mann–Kendall Tests for Trend Analysis in Water Resources Time Series. Advances in Time Series Methods and Applications.

[30] Coulibaly P., Baldwin C.K. (2005). Nonstationary hydrological time series forecasting using nonlinear dynamic methods. J. Hydrol..

[31] Khan S., Ganguly A.R., Bandyopadhyay S., Saigal S., Erickson D.J., Protopopescu V., Ostrouchov G. (2006). Nonlinear statistics reveals stronger ties between ENSO and the tropical hydrological cycle. Geophys. Res. Lett..

[32] Hao Z., Singh V.P. (2016). Review of dependence modeling in hydrology and water resources. Prog. Phys. Geogr..

[33] Fleming S.W., Dahlke H.E. (2014). Parabolic northern-hemisphere river flow teleconnections to El Niño-Southern Oscillation and the Arctic Oscillation. Environ. Res. Lett..

[34] Cannon A.J. (2015). Revisiting the nonlinear relationship between ENSO and winter extreme station precipitation in North America. Int. J. Climatol..

[35] Lin-Ye J., Garcia-Leon M., Gracia V., Ortego M.I., Lionello P., Sanchez-Arcilla A. (2017). Multivariate statistical modeling of future marine storms. Appl. Ocean Res..

[36] Kusumastuti D.I., Struthers I., Sivapalan M., Reynolds D.A. (2007). Threshold effects in catchment storm response and the occurrence and magnitude of flood events: Implications for flood frequency. Hydrol. Earth Syst. Sci..

[37] Konapala G., Veettil A.V., Mishra A.K. (2017). Teleconnection between low flows and large-scale climate indices in Texas River basins. Stoch. Environ. Res. Risk Assess..

[38] Mishra A.K., Özger M., Singh V.P. (2009). An entropy based investigation into the variability of precipitation. J. Hydrol..

[39] Mishra A.K., Özger M., Singh V.P. (2011). Association between uncertainty in meteorological variables and water resources planning for Texas. J. Hydrol. Eng..

[40] Mishra A.K., Coulibaly P. (2010). Hydrometric network evaluation for Canadian watersheds. J. Hydrol..

[41] Mishra A.K., Coulibaly P. (2014). Variability in Canadian Seasonal Streamflow Information and its Implication for Hydrometric Network Design. J. Hydrol. Eng..

[42] Li C., Singh V.P., Mishra A.K. (2012). Entropy theory-based criterion for hydrometric network evaluation and design: Maximum information minimum redundancy. Water Resour. Res..

[43] Mishra A.K., Singh V.P. (2009). Analysis of drought severity-area-frequency curves using a general circulation model and scenario uncertainty. J. Geophys. Res. Atmos..

[44] Mishra A.K., Özger M., Singh V.P. (2009). Trend and persistence of precipitation under climate change scenarios. Hydrol. Proc..

[45] Rajsekhar D., Mishra A.K., Singh V.P. (2013). Regionalization of drought characteristics using an entropy approach. J. Hydrol. Eng..

[46] Sharma A. (2000). Seasonal to interannual rainfall probabilistic forecasts for improved water supply management: Part 1—A strategy for system predictor identification. J. Hydrol..

[47] Harrold T.I., Sharma A., Sheather S. (2001). Selection of a kernel bandwidth for measuring dependence in hydrologic time series using the mutual information criterion. Stoch. Environ. Res. Risk Assess..

[48] Song X., Zhang J., Zhan C., Xuan Y., Ye M., Xu C. (2015). Global sensitivity analysis in hydrological modeling: Review of concepts, methods, theoretical framework, and applications. J. Hydrol..

[49] Han M., Ren W., Liu X. (2015). Joint mutual information-based input variable selection for multivariate time series modeling. Eng. Appl. Artif. Intell..

[50] Knuth K.H., Gotera A., Curry C.T., Huyser K.A., Wheeler K.R., Rossow W.B. (2013). Revealing relationships among relevant climate variables with information theory. arXiv.

[51] Khokhlov V.N., Glushkov A.V., Loboda N.S. (2006). On the nonlinear interaction between global teleconnection patterns. Q. J. R. Meteorol. Soc..

[52] Hurtado A.F., Poveda G. (2009). Linear and global space-time dependence and Taylor hypotheses for rainfall in the tropical Andes. J. Geophys. Res..

[53] Naumann G., Vargas W.M. (2010). Joint diagnostic of the surface air temperature in southern South America and the Madden–Julian oscillation. Weather Forecast.

[54] Yoon S., Lee T. (2016). Investigation of hydrological variability in the Korean Peninsula with the ENSO teleconnections. Proc. IAHS.

[55] Sivakumar B. (2016). Chaos in Hydrology: Bridging Determinism and Stochasticity.

[56] Varis O., Kummu M., Salmivaara A. (2012). Ten major rivers in monsoon Asia-Pacific: An assessment of vulnerability. Appl. Geogr..

[57] Wilmott C.J., Rowe C.M., Philpot W.D. (1985). Small-scale climate maps: A sensitivity analysis of some common assumptions associated with grid-point interpolation and contouring. Am. Cartogr..

[58] Yatagai A., Kamiguchi K., Arakawa O., Hamada A., Yasutomi N., Kitoh A. (2012). APHRODITE: Constructing a long-term daily gridded precipitation dataset for Asia based on a dense network of rain gauges. Bull. Am. Meteorol. Soc..

[59] Vu M.T., Raghavan V.S., Liong S.Y. (2012). SWAT use of gridded observations for simulating runoff—A Vietnam river basin study. Hydrol. Earth Syst. Sci..

[60] Vu M.T., Mishra A.K. (2016). Spatial and Temporal Variability of Standardized Precipitation Index over Indochina Peninsula. Cuad. Investig. Geogr..

[61] Vu M.T., Raghavan S.V., Liong S.-Y., Mishra A.K. (2017). Uncertainties in gridded precipitation observations in characterizing spatio-temporal drought and wetness over Vietnam. Int. J. Climatol..

[62] Raghavan V.S., Vu M.T., Liong S.Y. (2017). Ensemble climate projections of mean and extreme rainfall over Vietnam. Glob. Planet. Chang..

[63] Raghavan V.S., Vu M.T., Liong S.Y. (2014). Impact of climate change on future stream flow in the Dakbla river. J. Hydroinform..

[64] Raghavan V.S., Liong S.Y., Vu M.T. (2012). Assessment of future stream flow over the Sesan catchment of the Lower Mekong Basin in Vietnam. Hydrol. Proc..

[65] Vu M.T., Aribarg T., Supratid S., Raghavan V.S., Liong S.Y. (2016). Statistical downscaling rainfall over Bangkok using Artificial Neural Network. Theor. Appl. Climatol..

[66] Trenberth K.E. (1997). The Definition of El Niño. Bull. Am. Meteorol. Soc..

[67] Trenberth K.E. The Climate Data Guide: Nino SST Indices (Nino 1+2, 3, 3.4, 4; ONI and TNI). https://climatedataguide.ucar.edu/climate-data/nino-sst-indices-nino-12-3-34-4-oni-and-tni.

[68] Moon Y.I., Rajagopalan B., Lall U. (1995). Estimation of mutual information using kernel density estimators. Phys. Rev. E.

[69] Granger C., Lin J.L. (1994). Using the mutual information coefficient to identify lags in nonlinear models. J. Time Ser. Anal..

[70] Joe H. (1989). Relative entropy measures of multivariate dependence. J. Am. Stat. Assoc..

[71] Khan S., Bandyopadhyay S., Ganguly A.R., Saigal S., Erickson D.J., Protopopescu V., Ostrouchov G. (2007). Relative performance of mutual information estimation methods for quantifying the dependence among short and noisy data. Phys. Rev. E.

[72] Kraskov A., Stögbauer H., Grassberger P. (2004). Estimating mutual information. Phys. Rev. E.

[73] Hull M.M.V. (2005). Edgeworth approximation of multivariate differential entropy. Neural Comput..

[74] Cellucci C.J., Albano A.M., Rapp P.E. (2005). Statistical validation of mutual information calculations: Comparison of alternative numerical algorithms. Phys. Rev. E.

[75] Silverman B.W. (1986). Density Estimation for Statistics and Data Analysis.

[76] Wand M.P., Jones M.C. (1994). Multivariate plug-in bandwidth selection. Comput. Stat..

[77] Ince R.A.A., Giordano B.L., Kayser C., Rousselet G.A., Gross J., Schyns P.G. (2017). A statistical framework for neuroimaging data analysis based on mutual information estimated via a Gaussian Copula. Hum. Brain Mapp..

[78] Räsänen T.A., Kummu M. (2013). Spatiotemporal influences of ENSO on precipitation and flood pulse in the Mekong River Basin. J. Hydrol..

[79] Xu Z.X., Takeuchi K., Ishidaira H. (2003). Correlation between El Niño–Southern Oscillation (ENSO) and precipitation in Southeast Asia and the Pacific region. Hydrol. Proc..

[80] Chen D., Cane M.A., Kaplan A., Zebiak S.E., Huang D. (2004). Predictability of El Niño over the past 148 years. Nature.

[81] Ludescher J., Gozolchiani A., Bogacheva M.I., Bunde A., Havlin S., Schellnhuber H.J. (2014). Very early warning of next El Niño. Proc. Natl. Acad. Sci. USA.

[82] Power S., Delage F., Chung C., Kociuba G., Keay K. (2013). Robust twenty-first-century projections of El Niño and related precipitation variability. Nature.

[83] Cai W., Borlace S., Lengaigne M., Van Rensch P., Collins M., Vecchi G., Timmermann A., Santoso A., McPhaden M.J., Wu L. (2014). Increasing frequency of extreme El Niño events due to greenhouse warming. Nat. Clim. Chang..

[84] Zhang G., Su X., Singh V.P., Ayantobo O.O. (2017). Modeling NDVI using Joint Entropy method considering hydro-meteorological driving factors in the middle reaches of Hei river basin. Entropy.

